# Smoking, dementia and cognitive decline in the elderly, a systematic review

**DOI:** 10.1186/1471-2318-8-36

**Published:** 2008-12-23

**Authors:** Ruth Peters, Ruth Poulter, James Warner, Nigel Beckett, Lisa Burch, Chris Bulpitt

**Affiliations:** 1 Experimental Medicine and Toxicology Imperial College Faculty of Medicine, Hammersmith campus Du Cane Road, London, W12 0NN, UK; 2 St Charles Hospital Exmoor Street, London, W10 6DZ, UK

## Abstract

**Background:**

Nicotine may aid reaction time, learning and memory, but smoking increases cardiovascular risk. Cardiovascular risk factors have been linked to increased risk of dementia. A previous meta-analysis found that current smokers were at higher risk of subsequent dementia, Alzheimer's disease, vascular dementia and cognitive decline.

**Methods:**

In order to update and examine this further a systematic review and meta-analysis was carried out using different search and inclusion criteria, database selection and more recent publications. Both reviews were restricted to those aged 65 and over.

**Results:**

The review reported here found a significantly increased risk of Alzheimer's disease with current smoking and a likely but not significantly increased risk of vascular dementia, dementia unspecified and cognitive decline. Neither review found clear relationships with former smoking.

**Conclusion:**

Current smoking increases risk of Alzheimer's disease and may increase risk of other dementias. This reinforces need for smoking cessation, particularly aged 65 and over. Nicotine alone needs further investigation.

## Background

That smoking has a negative impact on health is no longer queried. In addition to the well known risks of lung cancer, smoking is also an independent predictor of cardiovascular morbidity, mortality and development of myocardial infarction [1]. Smoking may also accelerate cerebral atrophy, perfusional decline and white matter lesions [2]. In contrast, nicotine has plausible mechanisms for aiding cognitive function. Throughout the cholinergic system there are nicotinic acetylcholine receptors which can bind to nicotine. Use of nicotine as an agonist is said to up regulate these receptors in a dose dependent fashion, possibly by several hundred percent, depending on brain region [3,4]. The use of nicotine has also been found to aid attention, reaction time and some learning and memory [4]. Although this effect may be short lived if exposure is discontinued, potential benefits may be conferred as such receptors are thought to decline with ageing, with Alzheimer's disease and Lewy Body dementia [4]. A histopathological study in this area found mixed results with a possible protective effect of smoking against senile plaque formation in twenty eight matched pairs, and a positive correlation between the amount of smoking and neurofibrilliary change, but in smokers only [5].

A Cochrane review attempted to investigate the use of nicotine in Alzheimer's disease, but found no suitable data [6]. Other authors have compared smokers and non smokers in case control studies of Alzheimer's disease or cross sectional population studies and found mixed results. For example, studies have found no difference between smokers and non smokers in white or African American populations [7]; significantly increased risk of current smoking (OR2.33) in a Chinese population [8] and a reduced risk in male smokers only, (but in an unmatched case control study) [9]. Still further studies found male and female differences [10], an association between current smoking and reduced psychomotor speed, cognitive flexibility [11] and no difference between smokers and non smokers aged over fifty years for memory, reasoning and simple choice reaction time [5,12]. The smokers however, did die earlier.

One of the difficulties with research in this area is the potential overlapping of risk and protective factors as well as the undoubtedly negative constituents in cigarette smoke, despite smoking remaining the primary means by which people gain nicotine. Other issues include the clustering of risk or protective factors, the possible cognitive protection from a healthy diet [13], the influence of drinking alcohol and obesity. One study found that smoking was associated with reduced risk of Alzheimer's disease in drinkers only [14,15], and in seventy year olds both smoking and obesity were associated with poorer cognition on a neuropsychological battery [16]. Smoking behaviour may also change over time and by sector or cohort within the population; for example, smoking rates may be markedly lower with increasing age, lower in women [17] and may be impacted upon by ill health or change in circumstances. Studies may also vary in quality with both matched and unmatched case control studies and much cross sectional data in the literature. In addition to this subjects, either non smokers or smokers, may self select for studies [18] and survival biases may be present with smokers dying younger [19].

As smoking may have a negative effect on the cardiovascular system, it may plausibly impact on the development of dementias, both Alzheimer's Disease and vascular dementia. Several issues combine to make the study of different dementias and smoking important, the impact of these dementias in society, the difficulty in identifying pure forms of either, the increased risk of dementia and cognitive decline in the elderly and the ageing population. All of this combines to focus on the need for an increased understanding of risk and protective factors, particularly if these can be impacted upon via public health messages.

The most recent systematic review is wide ranging and was published by an Australian group in June 2007. The authors examined data from the start of the literature databases 'Pubmed', 'Psychinfo' and 'Cochrane CENTRAL' to June 2005 [20]. It focused on those over the age of 65 which is pertinent since the risk of dementia increases strikingly with increasing age. This review included only longitudinal studies and found that current smokers relative to never smokers were at increased risk of Alzheimer's disease, Vascular dementia, any dementia and cognitive decline. Former smokers had lower risks than current smokers at least for Alzheimer's disease and cognitive decline [20]. In parallel to their observations we carried out a similar systematic review using narrower search terms, wider inclusion criteria and more modern literature, including longitudinal studies published between 1995 and 2007 and identified using the literature databases Embase, Psychinfo and Medline. Our review was aimed to examine the relationship between smoking, dementia and cognitive decline in an elderly population.

## Method

Search terms 'smoking' and 'dementia' or 'vascular dementia' or 'multi infarct dementia' or 'Alzheimer's disease' or 'cognitive impairment' or 'cognitive decline' were used as keywords. The databases Medline, Embase and Psychinfo were searched for English language publications relating to human populations and which occurred between 1996 and November 2007. The Cochrane database of reviews was also searched. This time period was chosen as the methodology and computing power available for research changed greatly in the early 1990s. When available, standard search categories were also used as matched to the above terms. All searches were limited to the pre-defined search engine limits of subjects aged 65 and over. Two health service researchers appraised all abstracts and selected manuscripts independently, any discrepancies in decisions were discussed to achieve a unanimous choice of articles. No hand searching was carried out.

Studies were quality assessed by both researchers in accordance with the guidelines presented by Stroup et al for the reporting of meta-analyses of epidemiological studies [21]. There were no randomised controlled trials. Case studies, letters, consensus opinion from conferences and expert opinions or editorials were not included. In order to aid investigation of causality only longitudinal studies were assessed. Where studies had multiple publications and their outcomes differed all papers were included. When the outcomes were the same the paper with the longest follow up was selected, in order to prevent doubly including data. Where possible, meta-analyses and funnel plots were created. The most conservative results were used from each of the constituent populations, because the different populations were heterogeneous and had received adjustment for different factors no further adjustment was carried out. The definitions of current, ex and never smokers were taken from the published papers.

## Results

50 relevant papers were selected for full text examination from the three search engines and 28 retained [22-49] (see additional file 1 for further details). The 28 papers described 23 longitudinal studies. These studies were extracted into a summary table, see additional file 2. Those where data were reported as Odds Ratios (OR) or Relative Risks (RR) were combined into meta-analyses.

❖ Five studies found a significant link between current smoking and increased risk of incident dementia [22-28].

❖ Seven studies found a significant link with increased risk of cognitive decline [29-32,43,46,49]

❖ Six found no significant link with dementia or cognitive decline and current smoking [34,36,37,39,40,42]

❖ One found a significant link between dementia and past or current smoking [47].

❖ No studies found a significant link between former smoking and incident cognitive decline or dementia.

❖ Of the other studies, one found an association between smoking and a diminished risk of decline for attention and visuospatial tasks [44] and one, that a lack of smoking significantly predicted maintenance of cognitive function, OR 1.73 (95% confidence interval 1.30–2.20) [48].

Two sets of meta-analyses were carried out. The summary ratios below are for current smokers against never or non-smokers for the four outcomes of

❖ Alzheimer's disease (1.59 (95% CI 1.15–2.20))

❖ vascular dementia (1.35 (95% CI 0.90–2.02))

❖ dementia unspecified (1.16 (95% CI 0.90–1.50))

❖ cognitive decline (1.20 (95% CI 0.90–1.59)),

For ex smokers compared to never smokers

❖ Alzheimer's disease (0.99 (95% CI 0.81–1.23))

❖ vascular dementia (1.05 (95% CI 0.72–1.54))

❖ dementia unspecified (0.90 (95% CI 0.75–1.07))

❖ cognitive decline (0.90 (95% CI 0.74–1.10)).

Measurements of heterogeneity were not significant with the exception of that for Alzheimer's disease with current smoking (Cochran Q = 23.25, p = 0.0015).

**Figure 1:**
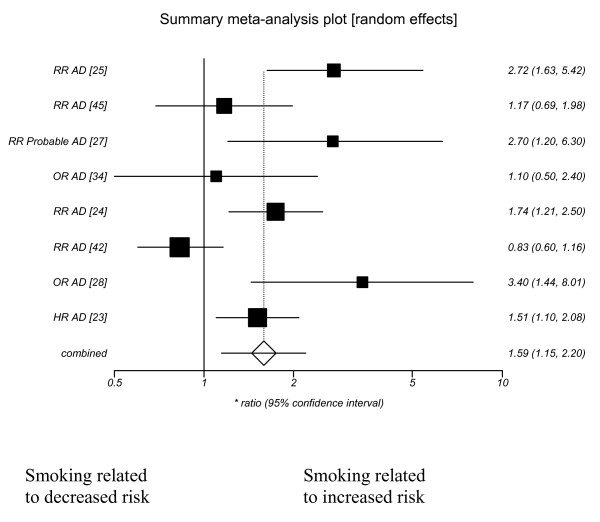
**Current smoking and Alzheimer's disease**.

**Figure 2:**
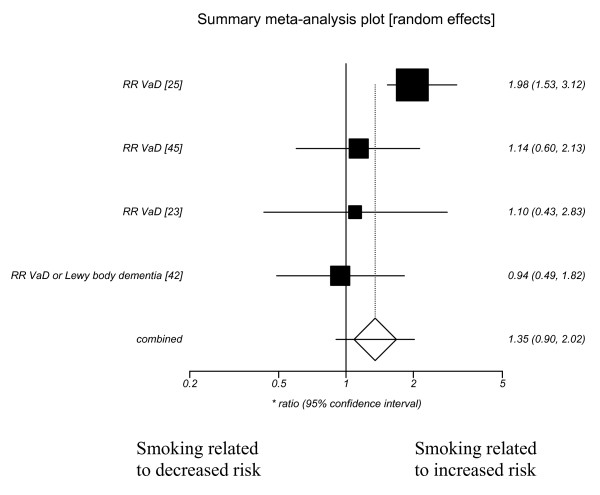
**Current smoking and vascular dementia**.

**Figure 3:**
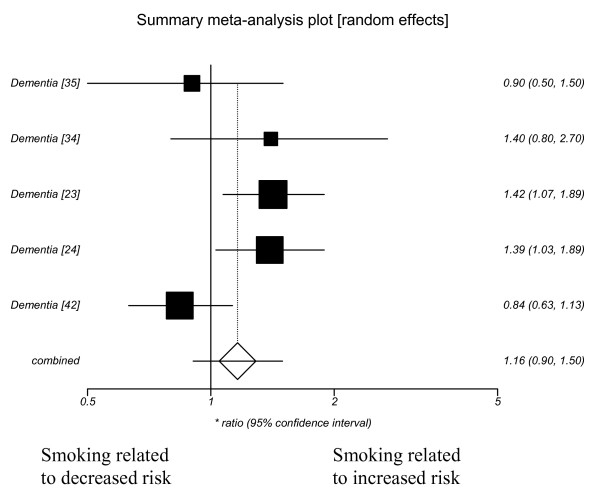
**Current smoking and dementia (unspecified)**.

**Figure 4:**
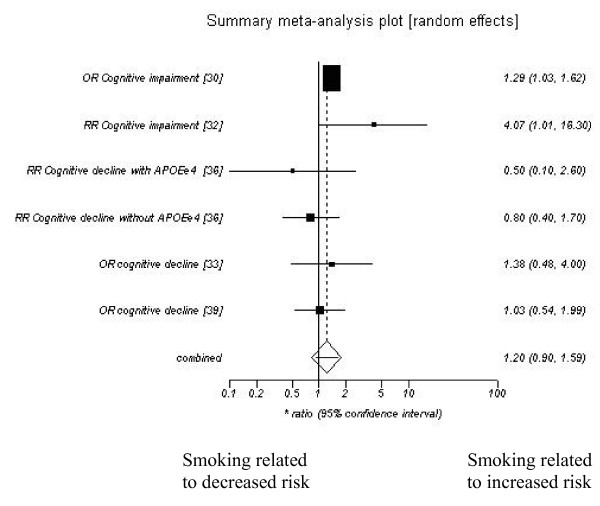
**Current smoking and cognitive decline**.

Publication bias did not seem evident from funnel plots (see additional file 3 for further details). Random effects models were used and the summary ratios showed a significantly increased risk of Alzheimer's disease with current smoking. Forest plots showing the effect of current smoking on Alzheimer's disease, Vascular dementia, dementia and cognitive decline are included as figures 1, 2, 3, 4.

These findings have to be understood in the context of the small numbers of results available for meta analysis for some outcomes and methodological issues such as the likelihood of increased mortality of smokers. Methodological issues are discussed further below.

### Methodological issues

#### Study design

Study design varied with cohort and case control methodology, as did length of follow up and population; with population ranging from less than 1000 to over 34,000 [31-34,37,39-42,44,49] and including participants from North America, China, Australia and Europe. Follow up times ranged from one [32] to 47 [42] years although the majority were less than 10 years. More detail is provided in table 1.

Not all studies gave a clear indication of participant age at baseline with some reporting mean age, some minimum age and some age range. Despite this, when taking the available data regarding baseline age and follow up time into account mean age at follow up seems likely to be 65 or over. The factors that were adjusted for in the reported analyses also varied with most authors adjusting for age, gender and education, although a handful included wider factors such as ethnicity, income, social class, history or current cardiovascular or respiratory diseases, blood pressure and alcohol use [22-49]

#### Outcome

Four studies had multiple publications, the Honolulu Asia Aging Study [30,45], the Washington Heights Medicare recipient's study [[26,27], and [31]], the Sydney Older Persons study [40,41] and the Rotterdam study [22,23]. Only those from the Sydney Older Persons study and the Rotterdam study had the same outcomes in each paper. The longest follow up was used in each case. Of the two publications from the Washington Heights data set, the cohort analysis reporting possible or probable Alzheimer's disease was used in preference to the case control analysis incorporating Alzheimer's disease. Each paper from the Honolulu Asia Aging study reported on a different outcome.

The majority of studies specified that prevalent dementia was excluded at baseline or that they had assessed incident dementia or cognitive decline [22-36,39-41,44-47]. The remainder reported study designs that made prevalent dementia at baseline highly unlikely. One study assessed smoking behaviour in midlife so although assessment for dementia in later life included prevalent cases these are highly unlikely to have been present at the time of smoking assessment [45]. Standardised criteria for the assessment and diagnosis of dementia and cognitive decline were used in most studies including those from the Diagnostic statistical Manual edition three revised (DSMIIIR) the National Institute of Neurological and Communicative Diseases and Stroke/Alzheimer's Disease and Related Disorders Association [NINCDS-ADRDA] for the diagnosis of Alzheimer's disease and the Mini-Mental State Exam (MMSE), [24,25,27,29,33,34,37,40,42,47,48]. Some studies used more than one instrument and some reported detailed neuropsychological testing [38,40,42,49]. Other instruments included the Portable Mental Status Questionnaire [39], the Clinical Dementia Rating scale [26,27], the Organic Brain syndrome scale [32] and the Cognitive Abilities Screening Instrument (CASI) [30,45]. One study used solely the information from death certificates [42].

The assessment of smoking was by self report and was also fairly standard with most studies reporting on smokers versus non smokers. Only cigarette use was reported in sufficient detail. Few studies reported the characteristics of smokers compared to non smokers but those who did found that never smokers were older, more highly educated, drank less alcohol and may be more likely to be female [25,29,30,34,45]. One study reported that former and current smokers showed younger age of dementia onset [22]. Unsurprisingly, mortality during study follow up was also greater with smoking [25,37,39,40,43,45] and three studies assessed causes of death from death certification data [22,34,42]. Non participation or loss to follow up was associated with lower educational levels, higher levels of impairment, age, smoking, body mass index, cardiovascular risk (for example diabetes and hypertension) and membership of an ethic minority group [36,38,39,44,46]. One study reported that those who refused to continue were not significantly different in age or MMSE score at baseline [40], another reported that they were older but there were no differences in tobacco use [36]. A further paper reported that those who refused to continue had lower MMSE and included proportionally more smokers and abstainers from alcohol [37] and another that lower income was associated with refusal but that there were no differences in follow up for sex, age, smoking or drinking levels [32]. Although the latter study followed subjects only for one year [32].

## Discussion

In comparison to the previous meta-analysis [20] which found an increased risk for all outcomes with current smoking (summary ratios and 95% confidence intervals); Alzheimer's disease 1.79 (1.43–2.23), vascular dementia 1.78 (1.28–2.47), any dementia 1.27 (1.02–1.60), our analyses found a significant relationship between increased risk of Alzheimer's disease and current smoking and summary ratios over one for vascular dementia, dementia unspecified and cognitive decline although they were not significant.

When we examined former smokers we found summary ratios close to unity for all outcomes and no significant results, as did the previously published meta-analysis using older literature. The meta-analysis reported here focused on the last 10 years of published literature up until November 2007 and included the database Embase whereas the most recently published analysis [20] omitted Embase but included 'Cochrane CENTRAL' and reported on older literature until June 2005. Although we chose to include more recent literature, less and different search terms and arguably wider inclusion criteria it is interesting to note that our findings are broadly in support of theirs and strengthening the conclusion that current smoking is likely to increase risk of incident dementia particularly Alzheimer's disease.

A note of caution should be sounded with regard to the interpretation of the data relating to exsmokers as the studies were not always consistent or clear with regard to the time since stopping smoking and the level of smoking that had previously been usual. Similar issues relate to current smokers in terms of amount, however, this was not consistently reported in the studies and may be biased by the higher death rates in smokers, likely to be even higher in heavy smokers. If pack years had been reported consistently in the published literature, or could be available for further analysis this may allow further exploration. Although only one of our meta-analyses was significant in measures of heterogeneity there remain methodological issues inevitably associated with combining several different studies and the flaws inherent in those studies themselves, in addition to this resource limitations did not permit hand searching and the published literature only was used without contacting individual authors directly or weighting individual studies. This is a limitation and may have resulted in an under-representation of negative studies and a possible source of bias. Despite these limitations it does seem likely that current smoking may be a risk factor for the most common dementia, Alzheimer's disease. It may also be a risk factor for other dementias and cognitive decline. These findings fit with other evidence of smoking as a risk factor for cardiovascular and cerebrovascular disease, stroke, silent infarction, increased oxidative stress, atherosclerosis and inflammation all of which may impact negatively on cognitive functioning and dementia incidence [50]. Evidence presented here adds another reason for ceasing to smoke or for preventing smoking from starting. The effects of nicotine itself may be different and at least one study found less decline in attentional processes with smoking which may be as would be expected from increased nicotine consumption. Further studies are clearly required although smoking is clearly not an advantageous way to test this.

## Conclusion

Despite the limitations inherent in combining multiple studies it seems likely from the analyses presented here that smoking increases risk of developing Alzheimer's disease and may also be a risk factor for other dementias. Certainly it provides a further reason for preventing or ceasing smoking.

## Competing interests

The authors declare that they have no competing interests.

Details of funding; none received and researchers therefore independent.

## Authors' contributions

All data gathering, analysis and writing was carried out by RPeters & RPoulter. CB, NB and JW provided advice and critical appraisal of the analyses to be performed, methods and writing style. LB reviewed and commented on the paper.

## Pre-publication history

The pre-publication history for this paper can be accessed here:



## Supplementary Material

Additional File 1 **Flow diagram showing papers selected by source.** Click here for file

Additional File 2 **An extraction table showing details of studies included**. Click here for file

Additional File 3 **Funnel plots for meta-analyses.** Click here for file
