# Supplementary material for: Smoking, dementia and cognitive decline in the elderly, a systematic review
Source: BMC Geriatr. 2008 Dec 23;8:36. doi: 10.1186/1471-2318-8-36 (PMC2642819; doi:10.1186/1471-2318-8-36)
Supplement: Additional File 1 — Flow diagram showing papers selected by source. [file 1471-2318-8-36-S1.doc]

Medline number identified 327

Psychinfo number identified 96

Medline number possibly relevant to research question 39 (judged on abstract alone)

Psychinfo number possibly relevant to research question 1 (judged on abstract alone)

Embase number possibly relevant to research question 35 (judged on abstract alone)

47 identified after overlap between search engines accounted for and full text examined.

23 rejected (inappropriate content or insufficient methodological detail)

25 papers retained

22 (longitudinal studies – 25 papers)

Embase number identified 328
