# Supplementary material for: Smoking, dementia and cognitive decline in the elderly, a systematic review
Source: BMC Geriatr. 2008 Dec 23;8:36. doi: 10.1186/1471-2318-8-36 (PMC2642819; doi:10.1186/1471-2318-8-36)
Supplement: Additional File 2 — An extraction table showing details of studies included. [file 1471-2318-8-36-S2.doc]

| Author | Population recruited from where | Baseline numbers, descriptives. Follow Up [FU] | Type of study | Main outcome cognitive function/dementia | Measurement of cognition or dementia | Relationship with smoking | Statistics if available.  OR=Odds Ratio, RR=Relative Risk, HR=Hazard Ratio, (numbers in brackets are 95% confidence intervals)  *In order to assess the most conservative finding, results used are those from the adjusted analyses unless otherwise stated.* |
| --- | --- | --- | --- | --- | --- | --- | --- |
| Juan D et al [25] | China 6 communities | N=2820 >=60 years  Prevalent cases excluded at baseline FU 2 years | Cohort | Dementia | MMSE, further cognitive testing & informant interview.  DSM-III-R used for diagnosis. | Current smoking significantly increased risk of all dementia types except ‘other’ Past smokers similar trend but not significant and higher smoking level increased risk – except for very heavy smokers | Relative risk of dementia subtypes by baseline cigarette smoking:  Compared to never smokers:  Past smokers:  RR AD 1.53 (0.65-1.42) **as written in the paper*  RR AD with CVD 1.61 (0.52-3.73)  RR AD without CVD 1.44 (0.61-2.26)  RR VaD 1.33 (0.51-3.02)  RR Other 1.75 (0.49-3.33)  Current smokers:  RR AD 2.72 (1.63-5.42)  RR AD with CVD 3.35 (1.33-4.71)  RR AD without CVD 2.39 (1.99-2.70)  RR VaD 1.98 (1.53-3.12)  RR Other 2.22 (0.39-6.19) |
| Wang et al [29] | China – 9 randomly selected communities | N=5437 enrolled >=55 years Mean FU 4.7 (SD 05) years | Cohort | Cognitive decline | MMSE | Current smoking may increase risk of later cognitive impairment | Of those who developed cognitive impairment 22.9% were current smokers at baseline, the corresponding value for those that remained cognitively intact was 18.5% p=0.009 No OR/RR/HR |
| Tyas et al [45] | Honolulu Asia aging study | N=3232, Age at baseline 58.6, SD 4.7 FU 25-30 years | Cohort  Japanese American men | Dementia | Cognitive ability screening instrument was used. Diagnosis of dementia was made on consensus conference using DSM-III-R AD was classified as probable or possib;le according to NINCDS-ADRDA. VaD was cladssified by California alzeimers diagnostic criteria | Current smoking and former smoking not significant. In smokers amount of smoking correlated with incident dementia of all types except for very heavy smokers. | Risk of Dementia by mid-life smoking status.  Former smokers compared to never smokers  OR AD 0.93 (0.58-1.50) OR AD+/- CVD 0.88(0.58-1.33)  OR VaD 0.82 (0.43-1.52)  OR all 0.80 (0.58-1.10)  Current smokers compared to never smokers  OR AD 1.17 (0.69-1.98) OR AD +/- CVD 1.31 (0.85-2.01)  OR VaD 1.14 (0.60-2.13)  OR all 1.11 (0.79-1.55) |
| Galanis et al [30] |  | N=3429 Age at baseline 58.6, SD 4.7. Cognitive assessment done 77.7 years, SD 4.6. FU 20-30 years |  | Cognitive decline | Cognitive ability screening instrument. | Continuous smoking is associated with higher risk than quitting or never smoking. Former smokers have a non significant increased risk. | Risk of cognitive impairment compared to never smokers.  Ex smokers OR 0.86 (0.68-1.07)  Quitters between exam 1 & 3 OR 1.30 (0.98-1.74)  Continuous smokers OR 1.29 (1.03-1.62)  Quitting at least 6 years before exam 3 (baseline) was associated with significantly lower risk compared to continuous smokers. |
| Merchant et al [26] | Medicare recipients from the community Washington Heights  Medicare recipients from the community Washington Heights | N=1062 baseline  Mean age of those who went on to develop AD 77.2 (SD5.7)  Those who did not go on to develop AD 75.1 (SD6.2)  Mean FU 2.04 years | Case control cohort | Alzheimer’s disease | AD diagnosed via examination by expert consensus and neuropsych testing and clinical dementia rating scale– based on standard criteria  AD only classified in the absence of all cerebrovascular disease | Current smoking significantly increased risk of AD | RR of Alzheimer’s disease smokers compared to never smokers  RR Past smokers: 0.7 (0.5-1.1)  RR Current smokers 1.7 (1.1-2.8) |
| Luchsinger et al [27] |  | Baseline >=65 years, at follow up 76.2 (5.9) years 69.8% female.  1138 (1012 with complete date) at baseline  Mean FU 5.5 years. |  | Probable, possible  Alzheimer’s  disease | Dementia diagnosis based on DSM-IV. AD diagnosed on NINCDS-ADRDA. | Smokers at significantly higher  risk of probable & possible AD – higher risk when combined with  other risk factors (diabetes, hypertension and  heart disease)  (41 participants were smokers) | Current smoking….  Probable and possible AD  HR 2.2 (1.0-4.9)  Probable AD  HR 2.7 (1.2-6.3) |
| Reitz et al [31] |  | N=791 baseline Mean age 75.6 (SD5.4) FU 5 years |  | Dementia and cognitive impairment (without dementia) | Screening via neuropsych battery, medical exam and dementia diagnosis via expert consensus and Clinical dementia rating scale | <75 years – no significant association between cognition and smoking  >75 years smokers perform more poorly in cognitive tests and memory declines faster in smokers without ApoeE4 as compared to never smokers or quitters | Current smokers was associated with faster cognitive decline only in memory among subjects aged >75year without ApoeE4 allele p=0.016  No OR/RR/HR |
| Cervilla et al [32] | Over 65 year olds recruited from the electoral ward of Gospel Oak, London | N=451 FU 1 year | Case control Cohort | Cognitive impairment | Organic brain syndrome scale (OBS), cognitive impairment scale from the short CARE structured assessment. | Current smokers were significantly more likely to be cognitively impaired when adjusted for smoking and drinking after age 65. | Controlling for smoking and drinking after age 65.  Ex smokers – adjusted result not clear in paper  Current smokers -RR 4.07 (1.01-16.3) |
| Wang et al [34] | Kungsholmen project Sweden. | N= 343, dementia free at baseline. Age over 75 years FU 3 years | cohort | All Dementia plus AD. | MMSE, diagnosis based on DSM-III-R. | Non significant increased risk for smokers. | Risk of smokers versus non smokers.  OR AD 1.1 (0.5-2.4)  OR Dementia 1.4 (0.8-2.7) |
| Yip et al [35] | MRC cognitive function and aging study, England and Wales | 4075 aged 65 and over. 3505 FU 2 and 6 years. | Nested case control study | dementia | Screened using MMSE. AGECAT algorithm, equivalent to DSM-III-R. | Past smokers in 2 years were significantly less likely to develop dementia. | Those who developed dementia during follow up:  Past smokers OR 0.7 (0.5-1.0)  Current smokers 0.9 (0.5-1.5) |
| Dufouil et al [36] | EVA (Epidemiology of vacular aging) study. Based in France | 1389 aged 59-71. 1094 FU 4 years | cohort | Cognitive decline | Defined by MMSE fall of 3 or more points between baseline and 4 years. | No significant findings – tending to lower relative risks | Compared to never smoker without APOE e4 Never smoker with APOE e 4 RR 1.1 (0.7-1.9)  Ex smoker without APOE e4 RR 1.1 (0.7-1.9) Ex smoker with APOE e4 RR 0.9 (0.4-2.0)  Current smoker without APOE e4 RR 0.8 (0.4-1.7) Current smoker with APOE e4 RR 0.5 (0.1-2.6) |
| Launer at al [37] | Zutphen Elderly study – Dutch contribution | Males 939 at baseline, 560 at FU after 5 years and 360 after 8 years age at baseline 65-84 years Average age at baseline 71.6 (SD 5.4), after 5 years 75.1 (4.7) after 8 years 78.7 (SD4.3) | Cohort  Dutch men | Cognitive decline | MMSE | No significant findings with pure scores but mean change in MMSE was significantly greater for all categories with CVD/diabetes | Mean change in MMSE only for longitudinal data |
| Knopman et al [38] | Atherosclerosis in communities cohort study ARIC | 14348 aged 45-64 at baseline 10963 at follow up Re-examined every 3 years –  6 year follow up (mean 6 – range 3.6-8.8) | Cohort | Cognitive decline | Neuropsychological battery including digit symbol substitution | Possible that decline in some neuropsychological tests is greater in smokers/exsmokers | Change in digit symbol substitution, delayed word recall and word fluency seem greater in current in former smokers – no detailed table with significxance values available but authors comment that older black subjects who were current smokers showed greater decline of the digit symbol substitutuion |
| Leibovici et al [44] | France general practitioners research network | 833 aged over 60 at baseline  Complete data at follow up 225 FU 3 years | Cohort | Cognitive decline – a drop of ten percent in a given cognitive function | Questionnaire on cognitive function (deterioration cognitive observee DECO) applied to all patients not meeting DSM-III criteria for dementia at baseline plus ECO computerised exam annually plus neurological exam in year 3 and semi structured interview and imaging | Smokers had a lower risk of decline on attention tasks | Smokers compared to the non smokers:  Diminished risk of decline for attention tasks OR 0.54(0.3-0.97) and visuospatial tasks OR 0.51(0.2-1.2)  Diminished risk of decline in visuospatial task reaction time OR 0.28(0.07-1.2) |
| Ford et al [39] | US | 647 at baseline, 529 in analysis for incident impairment. 74 years and over excluded prevalent cognitive impairment. FU 4 years | Cohort | Cognitive impairment | Portable mental status questionnaire. Cognitive impairment based on 4 or more errors on follow up visit as long as less than 4 on previous visit. | Not significant. | Overall compared to non smokers Smokers OR 1.03 (0.54-1.99) |
| Prince et al [43] | MRC General Practice Research Framework, UK | 2567 aged 65-74 years FU 54 months | Cohort | Cognitive function | Paired Associated Learning Test (PALT) and Trailmaking Test Part A (TMT) administered at entry, month 9, 21 and 54, Ravens Progressive Matrices (RM) and New Adult Reading Test (NART) administered at entry. | Associations between daily dose of cigarettes and decline in Paired Associate Learning Test score only significant in Females of lower than median intelligence. | No OR/RR/HR |
| Atkinson et al [33] | Baltimore, Maryland, older women | 558 participants, mean age 78 SD 8.1 years FU 3 years | Cohort | Cognitive decline | MMSE, decline defined as decrease to less than 24 in at least one annual follow-up visit. Gereatric depression Scale (GDS) also used. | Current smoking was significantly associated with combined decline -cognitive decline alone not significant. | Association between smoking and cognitive decline compared to never smoking:  Former smokers OR 1.03 (0.43-2.46)  Current smokers OR 1.38 (0.48-4.0) |
| Launer et al [24] | European studies – Denmark, France, the Netherlands and UK. | 16334 participants aged 65 years and older. Mean FU 2.25 years | Pooled analysis of cohort studies | Dementia | MMSE, Geriatric mental State exam & the Cambridge Examination of mental disorders cognitive test. High scorers then had detailed neuropsychological testing, an informant interview and a clinical examination. Used DSM-III-R and NINCDS-ADRDA criteria to diagnose possible and probable AD. | Current smoking significantly associated with increased risk of AD, and all dementias. | Risk of AD compared with Never smokers  Former smokers RR 1.19 (0.8-1.51)  Current smokers RR 1.74 (1.21-2.5)  Risk of all dementias compared with Never smokers  Former smokers RR 1.03 (0.79-1.34)  Current smokers RR 1.39 (1.03-1.89) |
| Ott et al [46] |  | 9209 participants aged 65 years and older. Mean FU 2.3 years |  | Cognitive decline | MMSE, neuropsychological testing, informant interview and a clinical examination | Smokers had significantly greater decline in MMSE score than those who never smoked. Significantly greater decline with higher cigarette pack year exposure. | Yearly mean change in MMSE compared to never smokers  Never smokers -0.03  Former smokers OR -0.06(-0.12—0.01)  Current smokers OR -0.16(-0.22—0.10) |
| Doll et al [42] | British doctors | 34439 males at baseline. 473 cases and 4 matched controls for each case used for analysis. FU 47 years. | Case control Cohort | Dementia | If stated on death certificate. 81 Were further assessed by comparing medical evidenced with the death certificate. Classified as AD if AD or presenile dementia stated on death certificate. Classified as Probable AD if Senile dementia or dementia stated on death certificate. Classified as Vascular dementia if Multiinfarct dementia or dementia with cerebrovascular disease stated on death certificate. | No significant relationship between dementia and smoking | Probable or definitely due to AD compared to never smokers  Continuing smokers RR 0.83 (0.60-1.16)  Ex smokers RR 0.78 (0.56-1.10)  Vascular or Lewy body compared to never smokers  Continuing smokers RR 0.94 (0.49-1.82)  Exsmokers RR 1.07 (0.57-2.02)  Any dementia compared to never smokers  Continuing smoker RR 0.85 (0.63-1.14)  Ex smokers RR 0.84 (0.63-1.13) |
| Lindsay et al [47] | Canadian study of health and aging | 4615 participants aged 65 years and over FU 5 years | Case control Cohort | Dementia | MMSE, at baseline screen positives (score below 78/100) and random sample of screen negatives then went on for a clinical examination and if then testable (scored 50+ in MMSE) a further neuropsychological testing. Case conference then diagnosed using DSM-III and NINCDS- ADRDA. At follow-up same procedure used except concensus conference diagnosed using DSM-IV as well as those used at baseline. | Smoking not significantly associated with AD | Association between smoking and risk of AD compared with controls  Cases OR 0.82 (0.57-1.17) |
| Piguet et al [40] | Sydney Older Persons Study | 377 subjects aged 75 and older. (mean age 80.4 +/-3.7 years) FU 6 years | Cohort | Incident dementia and cognitive decline | MMSE and other tests of language, memory, visuoperceptual and executive abilities. Dementia diagnosed using DSM-III-R critieria. Re-tested after 6 years. Then all information assessed by geriatrician and made a dementia diagnosis based on DSM-IV criteria. Possible or probable AD diagnosed based on NINDS-ADRDA criteria. | No significant association between smoking and dementia. | No OR or RR just means for smoking  (number of packets per day per year): |
| Broe et al [41] |  | 327 participants aged 75 years and older (mean age 83.4 years) FU 3 years |  | Dementia and cognitive decline | MMSE and medical and neurological test at baseline. Further battery of tests at 3 years. | Smoking was not a significant predictor of incident dementia | Partial correlations between smoking and cognitive test performance selected tests:  Clock drawing Current smokers: 0.06 Ex smokers: -0.05  MMSE Current smokers: 0.05 Ex smokers: 0.04 |
| Aggarwal et al [28] | Chicago Health and Aging Project (CHAP) | 1064 participants aged 65 years and older (mean age 73.8 years SD 9.6) Mean FU 4 years | Cohort | Incident AD | Clinical evaluation, neurological exam including a battery of tests. Clinician assessed all information and made diagnoses of AD based on NINCDS-ADRDA. NINDS-AIREN criteria was used for vascular dementia. | Risk of incident dementia significantly higher for current smokers compared to non smokers. Only current smokers with no e4 allele significantly at risk. No significant risk for Former smokers. | Smoking status and incident AD compared to never smokers.  Current smokers OR 3.40 (1.44-8.01)  Former smokers OR 0.90 (0.47-1.70) |
| Reitz et al [23] | Rotterdam study | 6868 aged 55 or older Mean FU 7.1 years | cohort | AD, VaD or dementia | MMSE & GMS. MMSE <26 or GMS organic level >0 then CAMDEX used. Suspected cases examined by neurologist and neuropsychologist and MRI if possible. Diagnosis of AD and dementia based on DSM-III-R and NINCDS-ADRDA criteria. For VaD used NINDS-AIREN criteria. | Current smokers without APOEe4 were more likely to develop any dementia and AD. There was no increased risk of VaD or for past smoking with any outcome | All subjects  Risk of dementia compared to never smokers.  past smokers HR 1.17 (0.92-1.48)  Current smokers HR 1.42 (1.07-1.89)  Risk of AD compared to never smokers.  past smokers HR 1.17 (0.90-1.52)  Current smokers HR 1.51 (1.10-2.08)  Risk of VaD compared to never smokers.  past smokers HR 1.26 (0.59-2.68)  Current smokers HR 1.10 (0.43-2.84)  Without APOEe4  Risk of dementia compared to never smokers.  past smokers HR 1.04 (0.73-1.49)  Current smokers HR 1.06 (0.67-1.69)  Risk of AD compared to never smokers.  past smokers HR 1.08 (0.73-1.60)  Current smokers HR 1.06 (0.62-1.79)  Risk of VaD compared to never smokers.  past smokers HR 1.59 (0.49-5.12)  Current smokers HR 0.84 (0.14-4.93) |
| Ott et al [22] |  | 6870 aged 55 or older Mean FU 2.1 years | cohort | AD, VaD or dementia. | MMSE, Gereatric mental state, clinical exams and DSM-III-R. Classified people with AD as with or without cerebrovascular disease. Age of onset of dementia was mid point between baseline age and age of diagnosis. Additional information for people who could not be examined was gained through medical records. | Current smokers with AD with and without CVD are significantly more likely to develop dementia. Former and current smokers were more likely to have dementia overall. Smoking had no effect with APOE e4 allele. | Risk of dementia compared to never smokers.  All subjects  Former smokers RR 1.4 (0.9-2.0)  Current smokers RR 2.2 (1.3-3.6)  AD without CVD  Former smokers RR 1.4 (0.8-2.3)  Current smokers RR 2.1 (1.1-4.0)  AD with CVD  Former smokers RR 1.2 (0.4-4.2)  Current smokers RR 3.9 (1.0-15.2)  VaD  Former smokers RR 1.4 (0.5-4.4)  Current smokers RR 2.2 (0.6-8.4)  Other dementia  Former smokers RR 1.5 (0.5-4.2)  Current smokers RR 2.1 (0.6-6.8) |
| Starr et al [49] | Scottish Mental Survey participants resident in Aberdeen at the time of original testing | 289 aged 66 FU 2 years | cohort | Neuropsychological testing with memory and information processing – cognitive decline | The Raven’s standard progressive matrices. Reys Auditory Verbal learning test. Uses of common objects test. Digit symbol subtest of the wechsler adult intelligence scale - Revised (WAIS-R). The block design sub test of WAIS-R. | Found an adverse effect of smoking on information processing speed and memory | Compared to current smokers, nonsmokers scored a mean of 4.9 points higher for memory testing and 2.6 for information processing, ex-smokers 3.5 points higher for memory testing and 1.9 for information processing. Smoking contributed significantly to lower performance on the Auditory Verbal Learning Test (p<0.001) and the Digit Symbol test (p=0.026) |
| Barnes et al [48] | The study of Osteoporotic Fractures | 9704 aged 65 and older, mean age 72 at baseline, FU 15 years – visits at 6,8,10,15 years after baseline | cohort | cognitive decline | MMSE | Lack of smoking predicted maintenance of optimal cognitive function | Maintenance of optimal cognitive function compared to minor cognitive decline; OR 1.73 (1.30-2.20) |
