# Supplementary material for: Smoking, dementia and cognitive decline in the elderly, a systematic review
Source: BMC Geriatr. 2008 Dec 23;8:36. doi: 10.1186/1471-2318-8-36 (PMC2642819; doi:10.1186/1471-2318-8-36)
Supplement: Additional File 3 — Funnel plots for meta-analyses. [file 1471-2318-8-36-S3.doc]

Cochran’s Q and funnel plots for Alzheimer’s disease and current smoking

Non-combinability of studies

Cochran Q = 23.250846 (df = 7) P = 0.0015

Cochran’s Q and funnel plots for vascular dementia and current smoking

Non-combinability of studies

Cochran Q = 5.417736 (df = 3) P = 0.1436

Cochran’s Q and funnel plots for dementia unspecified and current smoking

Non-combinability of studies

Cochran Q = 9.171697 (df = 4) P = 0.0569

Cochran’s Q and funnel plots for cognitive decline and current smoking

Non-combinability of studies

Cochran Q = 5.874359 (df = 5) P = 0.3186

Cochran’s Q and funnel plots for Alzheimer’s disease and ex-smokers

Non-combinability of studies

Cochran Q = 11.269089 (df = 6) P = 0.0804

Cochran’s Q and funnel plots for vascular dementia and ex-smokers

Non-combinability of studies

Cochran Q = 1.344066 (df = 3) P = 0.7187

Cochran’s Q and funnel plots for dementia unspecified and ex-smokers

Non-combinability of studies

Cochran Q = 3.864438 (df = 3) P = 0.2765

Cochran’s Q and funnel plots for cognitive decline and ex-smokers

Non-combinability of studies

Cochran Q = 0.865691 (df = 3) P = 0.8337
